# Single Nucleotide Polymorphisms of Growth Hormone and Insulin‐Like Growth Factor‐1 Genes and Their Association With Growth Traits of Goats: A Systematic Review

**DOI:** 10.1002/vms3.70998

**Published:** 2026-05-18

**Authors:** Thlarihani Cynthia Makamu, Thobela Louis Tyasi, Monnye Mabelebele

**Affiliations:** ^1^ Department of Agriculture and Animal Health, College of Agriculture and Environmental Sciences University of South Africa Pretoria Gauteng South Africa; ^2^ Department of Agricultural Economics and Animal Production University of Limpopo Sovenga Limpopo South Africa

**Keywords:** body conformation, body measurements, body weight, genetic polymorphisms, small stock

## Abstract

*Insulin‐like growth factor‐1* (*IGF‐1*) and *growth hormone* (*GH*) genes are vital for animal growth. This study aimed to review the single nucleotide polymorphisms (SNPs) of *IGF‐1* and *GH* genes and their associations with growth traits of goats. This study is in accordance with the Preferred Reporting Items for Systematic Reviews and Meta‐Analyses (PRISMA). Databases (Google Scholar, PubMed, ScienceDirect and Web of Science) were evaluated. Eight SNPs of the *IGF‐1* gene were found in the selected studies, whereas nine SNPs of *GH* gene were discovered. One article found two SNPs (1617 G>A and 5752 G>C) that were similar to the ones of two other articles, each for the *GH* gene, and these articles found that body weight (BW) was not associated with the SNPs. All the other articles discovered different SNPs from each other for both genes. Four of the articles found an association between *GH* genotypes and BW. One article discovered a relationship between *GH* genotypes and withers height and chest width (CW) and three articles found an association between *GH* genotypes and heart girth (HG). Four articles discovered an association between *IGF‐1* genotypes and BW and one article discovered it with CW. This study concluded that the *IGF‐1* gene might be used as a potential molecular marker for the genetic improvement of BW and CW in goat breeding, whereas the *GH* gene can be used for the genetic improvement of BW, withers height, HG, body length and CW in goat breeding.

## Graphical abstract

This study found that *insulin‐like growth factor‐1 (IGF‐1) and growth hormone (GH)* genes were vital for animal growth and might be used as a potential molecular marker for the genetic improvement of growth traits in goats. Four of the articles found an association between *GH* genotypes and BW. Four articles discovered an association between *IGF‐1* genotypes and BW and one article discovered it with CW.

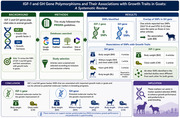

## 1 Introduction

Throughout the world, goat breeding is essential to agriculture and provides a substantial income for millions of people, particularly in poorer nations (Ayele et al. 2024). Goats have been reported to have low growth performance, making it necessary for genetic improvement of growth traits (Mokoena et al. 2023; Ncube et al. 2022). Low productivity of goats may be due to different factors such as lack of appropriate breed and breeding strategies (Deribe et al. 2015), thus making it imperative to understand and improve the growth performance of these animals. Growth traits have long aroused interest in the production of meat animals (Lestari et al. 2020). The early growth rate of goats affects profitability in goat production (Sutopo et al. 2021). It also has a strong implication for both reproductive and production performances because they are the basis for selection in genetic improvement programmes for meat production (Aradhana et al. 2021). With the development of molecular biology and biotechnology, scientists can achieve more accurate and efficient selection goals through marker‐assisted selection (Ayele et al. 2024). The physiological regulation of domestic animals’ growth is controlled by multiple genes, and the genetic variants of these genes may have associations with the economically relevant traits (Shareef et al. 2018). Polymorphisms in these candidate genes that show association with specific traits are useful quantitative trait nucleotides (Sharma et al. 2013). *Growth hormone* (*GH*) and *insulin‐like growth factor‐1* (*IGF‐1*) genes operate in coordination to regulate the growth performance of livestock (Angel et al. 2018). The *GH* gene influences the growth of bones and muscles, which is mediated by the *IGF‐1* gene, which is a key factor for the postnatal growth of goats.

All the articles included in this research provide different conclusions on the effect of *IGF‐1* and *GH* genes on growth traits of goats. Hence, this systematic review integrates existing findings and gives a summarized conclusion regarding the possibility of these genes being used as genetic markers. This review summarizes the regions of the SNPs of *IGF‐1* and *GH* genes, allelic and genotypic frequencies, and the SNPs association with growth traits of goats, since to the best of our knowledge, there is no systematic review providing the above‐mentioned information. This systematic review will help researchers and small ruminant farmers to know the association that single nucleotide polymorphism (SNP) of *GH* and *IGF‐1* genes have with growth traits of goats. This study will also aid in highlighting and detailing the recent developments and discoveries regarding the SNPs of *IGF‐1* and *GH* genes and their potential use as genetic markers in goats for marker‐assisted selection during breeding. This study aimed to provide information on the effect of *IGF‐1* and *GH* genes and their associations with the growth traits of goats.

## 2 Materials and Methods

### 2.1 Eligibility Criteria

To undertake a systematic review, it is necessary to identify the population, exposure and outcomes (PEO) components of the research question as explained by Bettany‐Saltikov (2010). The population was defined as ‘Goats’, with an exposure of ‘Polymorphisms’ and outcomes of ‘Body Measurement traits’. A preliminary search of the PEO components on Google Scholar, Web of Science, PubMed and Science Direct search engines was conducted before conducting the study.

### 2.2 Search Strategy for Identification of Relevant Studies

The current systematic review was conducted in accordance with the Preferred Reporting Items for Systematic Reviews and Meta‐Analyses (PRISMA) (Page et al. 2021). Google Scholar, PubMed, Science Direct and Web of Science search engines were used for publication search from October to November 2024. ‘Polymorphisms/single nucleotide polymorphisms/genetic polymorphisms/genetic effect/genetic variants’, ‘*growth hormone (GH)* gene/*insulin‐like growth factor‐1* (*IGF‐1*) gene’, ‘body measurement traits/growth traits/body conformation/body weight/morphological traits/biometric traits’ and ‘goats/small stock/small ruminants’ were used as key terms when the publication search was performed.

### 2.3 Inclusion Criteria

Titles and abstracts found using the search strategy were screened manually to identify studies that were potentially relevant. Studies were considered for inclusion in the systematic review provided: They are written in English, and they investigated the associations between growth traits with *GH* and *IGF‐1* genes in goats. Only articles that passed all the screenings for title, abstract and eligibility were included in this systematic review.

### 2.4 Exclusion Criteria

Articles that were written about a different species other than goats, missing one of the keywords, not clear on the association of the genes and growth traits, and those that were using genes other than *GH* and or *IGF‐1* genes were excluded. Duplicate studies were also excluded. Articles were screened for title, abstract and eligibility. Articles with missing keywords and those using different species in the title were excluded from this systematic review without screening the abstract. Those that passed screening for title were then screened for abstract, and the ones that could not pass screening for abstract were excluded. Articles that passed screening for abstract were then screened for eligibility; if they didn't pass eligibility, they were then excluded from this systematic review.

### 2.5 Data Extraction

The researchers extracted study content and data independently, and an agreement was reached concerning all key items. The content of the studies included the first author's name, year of publication, geographical location, goats, sample size and studied growth traits as shown in Table 1.

**TABLE 1:** Characterization of included studies.

Author/year	Country	Breed	Sample size	Growth traits	Genotyping method
Alex et al. 2023	India	Attappady Black, Malabari	100	BW	T‐ARMS‐PCR
An et al. 2010	China	Saanen, Boer	358	BW, WH, BL, HG	PCR‐SSCP
Ayele et al. 2024	Türkiye	Saanen, Alpine, Boer	203	BW, WH, BL, HG, CW	PCR‐RFLP
Hua et al. 2009	China	Boer	154	BW, BL, HG	PCR–RFLP
Kurdistani et al. 2013	United States of America	Markhoz, Kurdi	296	BW	PCR–RFLP
Rashijane et al. 2022	South Africa	Boer	76	BW, BL, HG, RH, RW, EL, CC, HW	PCR‐RFLP
Rasouli et al. 2017	Iran	Markhoz	152	BW	PCR‐SSCP
Sarmah et al. 2019	India	Assam Hill	256	BW	PCR‐RFLP
Shareef et al. 2018	Pakistan	Beetal	60	BW, WH, BL, HG	PCR‐RFLP
Sharma et al. 2013	India	Sirohi, Jamunapari	389	BW	PCR
Zhang et al. 2008	China	Nanjiang Huang	592	BW, BL, WH, HG	PCR‐SSCP

Abbreviations: BL = body length, BW = body weight, CC = cannon circumference, CW = chest width, EL = ear length, HG = heart girth, HW = head width, PCR = polymerase chain reaction, PCR‐RFLP = polymerase chain reaction‐restriction fragment length polymorphisms, PCR‐SSCP = polymerase chain reaction‐single‐strand conformation polymorphisms, RH = rump height, RW = rump width, T‐ARMS‐PCR = tetra‐primer amplification refractory mutation system‐polymerase chain reaction, WH = withers height.

### 2.6 Quality of Studies

Studies selected for this systematic review were assessed for quality of reporting and selection for bias using a quality assessment checklist (Ahaduzzaman 2020). The checklist included nine parameters that have ‘yes’ and ‘no’ applicable options. Operationally, the ‘yes’ answer was scored 1; while ‘no’ was provided with 0 scores. Ultimately, for each article, the mean score was determined. Studies were classified as follows: low quality = 0−3, moderate quality = 4−6 and high quality = 7−9.

## 3 Results

### 3.1 Searched Results

**FIGURE 1:**
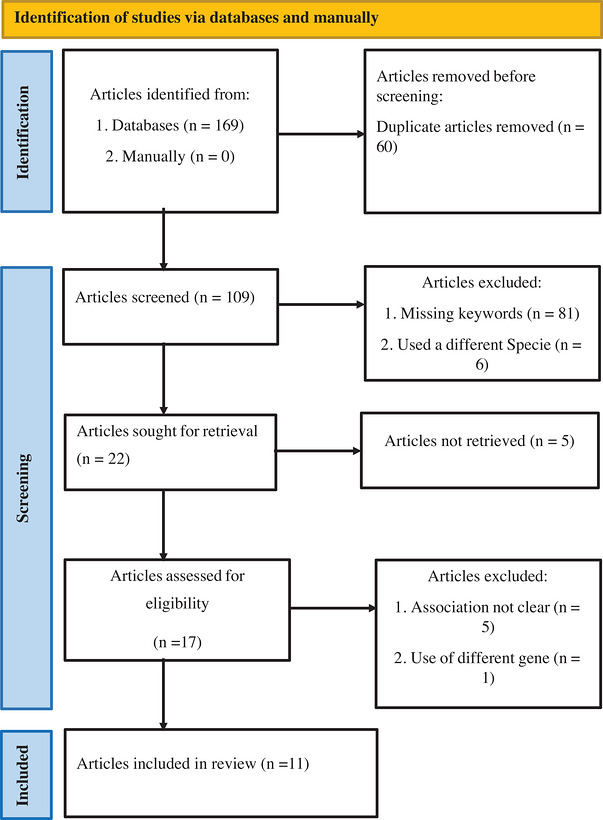
Literature search and selection process following the PRISMA procedure.

Articles that were identified in the electronic databases were 169 (*n* = 169), as shown in Figure 1 below. Studies that remained after the exclusion of duplicates were 109 (*n* = 109). About 87 (*n* = 87) more articles that did not fit our scope were eliminated when screening. Twenty‐two (*n* = 22) articles were left and 17 (*n* = 17) were retrieved; five (*n* = 5) of them were not retrieved. Among the 17 (*n* = 17) studies that were selected for full reading, six (*n* = 6) studies did not meet the eligibility criteria and they were excluded. Finally, a total of 11 (*n* = 11) studies were included in this systematic review.

### 3.2 Characteristics of Included Studies

Characteristics of 11 (*n* = 11) included studies are shown in Table 1. They were conducted in seven (*n* = 7) different countries. About 12 (*n* = 12) different breeds were studied in these selected articles. The sample sizes used differed per study, and they ranged from 76 to 592. About 10 (*n* = 10) different growth traits were studied. This table provides detailed, structured summary of each included study's key features which are essential for several reasons.

### 3.3 Publication by Country

**FIGURE 2:**
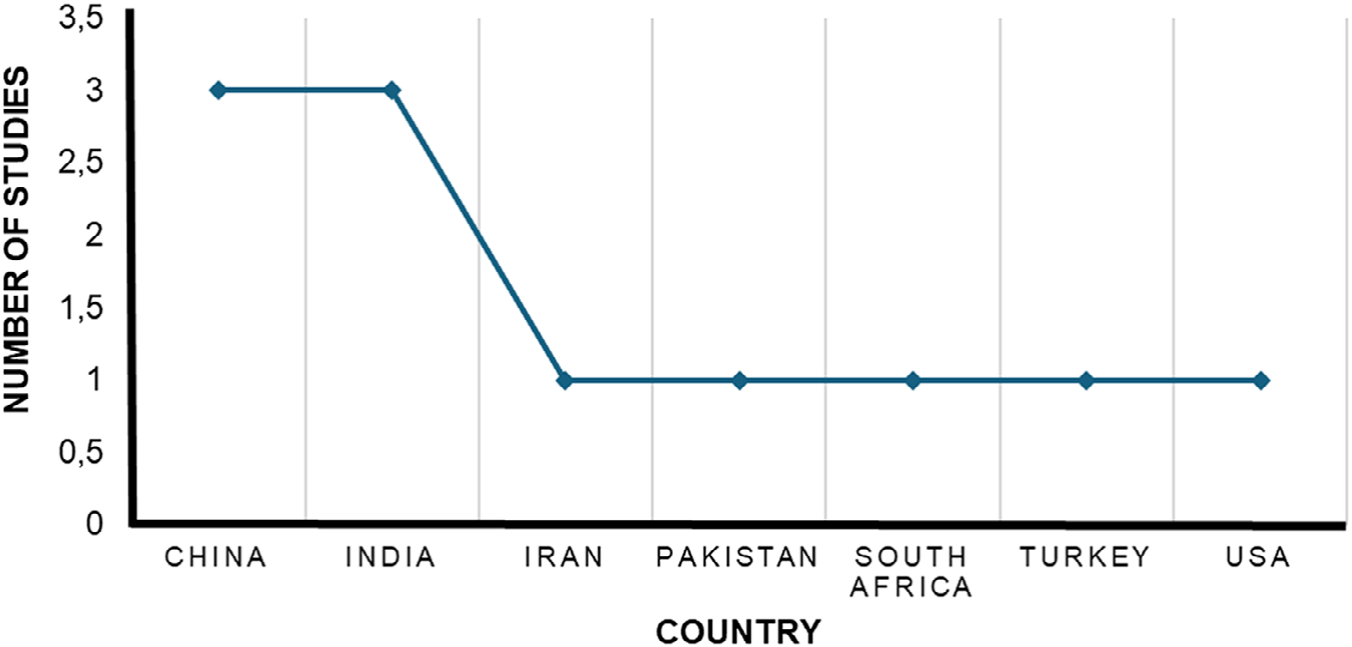
Publication by country.

The number of selected studies published by country is shown in Figure 2 below. The results showed that these studies were conducted in seven (*n* = 7) different countries, namely, India, China, Türkiye, United States of America, South Africa, Iran and Pakistan. India (Sharma et al. 2013; Sarmah et al. 2019; Alex et al. 2023) and China (Zhang et al. 2008; Hua et al. 2009; An et al. 2010) ranked first with three studies each. The other countries only published one article each. Most of the studies were conducted in Asia. Only two studies were conducted in two different continents which were Africa and America, meaning that the other four continents had no study.

### 3.4 Publication by Year

**FIGURE 3:**
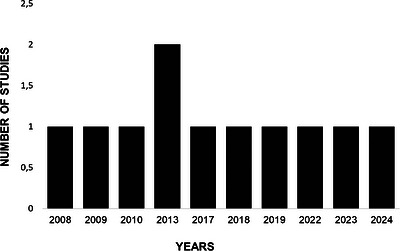
Publication by year.

Figure 3 below shows the year that the studies were published in. The findings revealed that the studies included were conducted from 2008 to 2024. Two (*n* = 2) studies were published in 2013, whereas the other nine (*n* = 9) studies were published in different years, with one study per year from 2008 to 2010, 2017 to 2019 and 2022 to 2024. All the studies were conducted in the 21st century, showing that from the 20th century going back, these kinds of studies were not conducted.

### 3.5 Distribution of Articles by Genotyping Method

**FIGURE 4:**
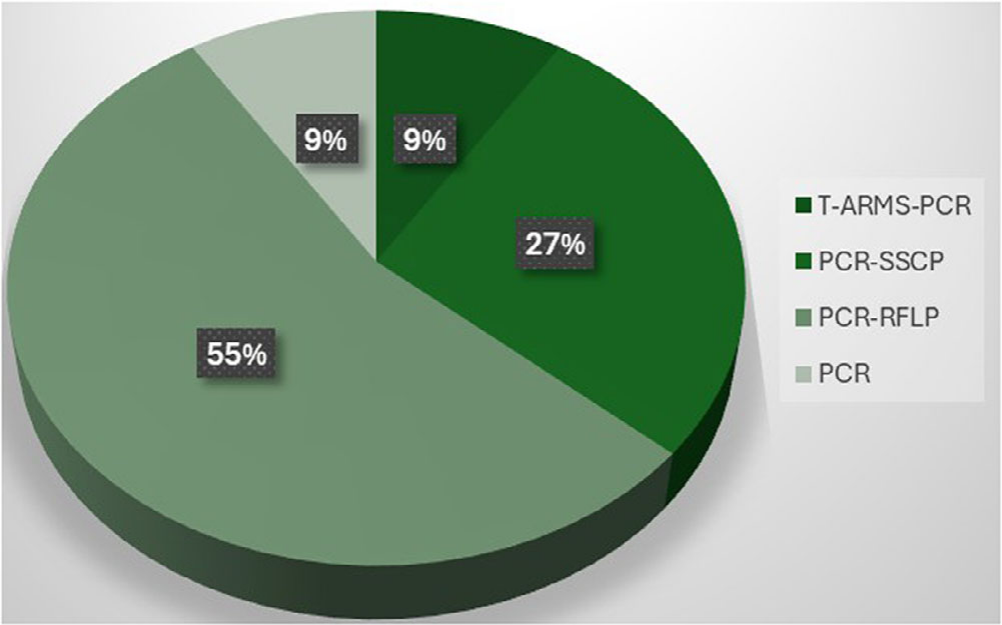
Publication by genotyping method.

The distribution of studies by genotyping methods are shown in Figure 4 below. About six (*n* = 6) articles used polymerase chain reaction‐restriction fragment length polymorphisms (PCR‐RFLP) as their genotyping method, whereas three (*n* = 3) used polymerase chain reaction‐single‐strand conformation polymorphisms (PCR‐SSCP) and one (*n* = 1) used tetra‐primer amplification refractory mutation system‐polymerase chain reaction (T‐ARMS‐PCR) (Alex et al. 2023). The study of Sharma et al. (2013) was not too clear about its genotyping method; however, it mentioned using polymerase chain reaction (PCR). Molecular (DNA) genetic analysis was the one used in the selected studies.

### 3.6 Distribution of Articles by Breed in GH Gene

**FIGURE 5:**
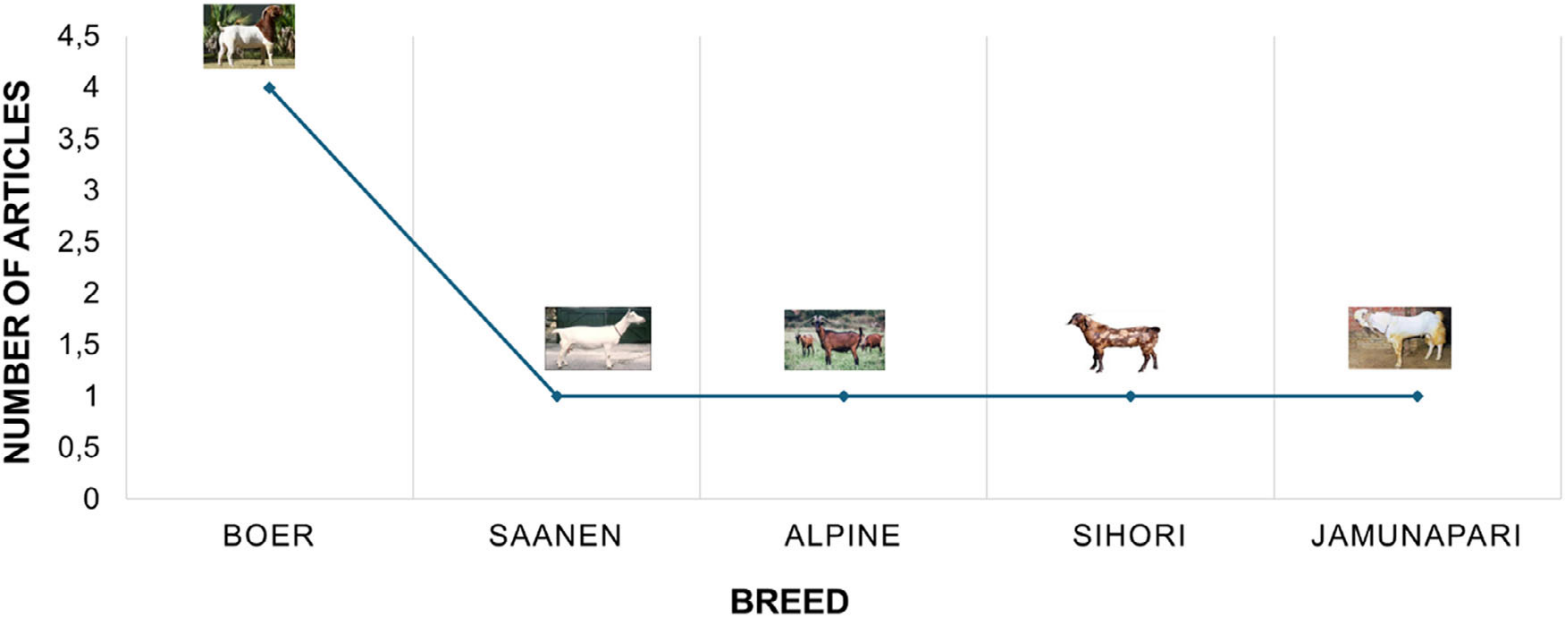
Allocation of articles by breed in *GH* gene.

The breeds used in the studies are shown in Figure 5 below. The results indicated that five (*n* = 5) different breeds were investigated for the association of growth traits and the *GH* gene. Four (*n* = 4) studies investigated the Boer goat breed. The other four (*n* = 4) goat breeds, namely, Saanen, Alpine, Sihori and Jamunapari were only investigated by one study each. The breeds studied included two dual‐purpose breeds, two milk and one meat purpose breed. Breeds that are bred for meat only were very low for the *GH* gene.

### 3.7 Distribution of Articles by Breed in IGF‐1 Gene

**FIGURE 6:**
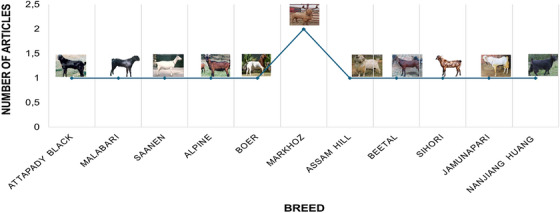
Distribution of articles by breed in *IGF‐1* gene.

Figure 6 below presents the allocation of articles by breed for *IGF‐1* gene. The results showed that 11 (*n* = 11) different goat breeds, namely, Attappady Black, Malabari, Saanen, Alpine, Boer, Markhoz, Assam Hill, Beetal, Sihori, Jamunapari and Nanjiang Huang were investigated for the association between *IGF‐1* gene and growth traits of goats. The most studied goat breed was found to be Markhoz that was studied by two (*n* = 2) articles, whereas all the other goat breeds were only studied by one article each. Most of the breeds used for this gene were meat purpose; there were few breeds used for dual purpose followed by milk then multipurpose goat breeds.

### 3.8 Identified SNPs and Regions

Table 2 below indicates the SNPs that were identified in the selected studies and their regions. The results indicated that six (*n* = 6) of the selected studies focused on *IGF‐1* gene whereas five (*n* = 5) of them focused on *GH* gene; one article (*n* = 1) (Sharma et al. 2013) focused on both *IGF‐1* and *GH* genes. Ten articles (*n* = 10) identified SNPs and nine (*n* = 9) of them also identified their regions. Most studies (Hua et al. 2009; Rasouli et al. 2017; Shareef et al. 2018) found their SNPs on Exon 2. Two (*n* = 2) articles per region found their SNPs on regions: Intron 1 and 2 and Exon 4 and 5. For other regions, only one article found its SNPs on each of them. Seven (*n* = 7) articles out of 10 (*n* = 10) discovered different SNPs from each other. Two SNPs that were discovered by Kurdistani et al. (2013) were similar to the ones discovered one by Rasouli et al. (2017) and the other by Sarmah et al. (2019). Most identified SNPs were different from each other, making it necessary for more studies to be conducted to discover similar SNPs and associate them with growth traits of goats.

**TABLE 2:** Single nucleotide polymorphisms (SNPs) and regions of *GH* gene and *IGF‐1* gene.

Author	Breed	*N*	Gene	Region	Identified SNPs
Alex et al. 2023	Attappady Black, Malabari	100	*IGF‐1*	Intron 2	c.546þ179170A>T
An et al. 2010	Saanen, Boer	358	*GH*	Exon 3 and 5	112A>G, 142C>T, 214C>T and 266C>A
Hua et al. 2009	Boer	154	*GH*	Exon 2 and 4	781A>G and 1575A>G
Kurdistani et al. 2013	Markhoz, Kurdi	296	*IGF‐1*	Intron 4 and Exon 4	1617 G>A and 5752 G>C
Rashijane et al. 2022	Boer	76	*GH*	Exon 5	505G>C
Rasouli et al. 2017	Markhoz	152	*IGF‐1*	Exon 2	1617 G>A
Sarmah et al. 2019	Assam Hill	256	*IGF‐1*	—	5752G>C
Shareef et al. 2018	Beetal	60	*GH*	Exon 2	825A>G
Sharma et al. 2013	Sirohi, Jamunapari	389	*IGF‐1*	Intron 1 and 2	4700T>C and 5524C>T
			*GH*	Promoter	188A>T
Zhang et al. 2008	Nanjiang Huang	592	*IGF‐1*	Intron 4	589G>C

### 3.9 Genotypic Frequencies for GH Gene

Genotypic frequencies from the reviewed articles of *GH* gene are shown in Table 3. Out of five (*n* = 5) articles that investigated association of growth traits with *GH* gene, only three (*n* = 3) were clear about their genotypic frequencies. One (*n* = 1) article discovered three genotypes per SNP, with two (*n* = 2) of the three genotypes shown per breed. Two (*n* = 2) genotypes were noted per SNP from two (*n* = 2) articles, with four (*n* = 4) of the two genotypes per breed. The genotypic frequencies ranged from 0.014 to 0.889. Most genotypes that showed high genotypic frequencies were homozygous, only two were heterozygous in the populations.

**TABLE 3:** Genotypic frequencies of *GH* gene.

Author	Breed	*N*	Genotypic frequencies
Ayele et al. 2024	Saanen	203	AB (0.876), BB (0.124)
	Alpine		AB (0.889), BB (0.111)
	Boer		AB (0.394), BB (0.606)
Rashijane et al. 2022	Boer	76	AA (0.79), AB (0.21)
Sharma et al. 2013	Sirohi	389	CC (0.840), TC (0.150), TT (0.006)
	Jamunapari		CC (0.770), TC (0.212), TT (0.014)

### 3.10 Genotypic Frequencies for IGF‐1 Gene

Genotypic frequencies from the reviewed articles of *IGF‐1* gene are shown in Table 4. Out of eight (*n* = 8) studies that investigated the association between *IGF‐1* gene and growth traits of goats, only seven (*n* = 7) of them were clear about their genotypic frequencies. All articles discovered three genotypes per SNP with 11 (*n* = 11) of the three genotypes shown per breed. The genotypic frequencies ranged from 0 to 0.97. All the genotypes that had high genotypic frequencies were homozygous. All the heterozygous ones were low in the investigated populations.

**TABLE 4:** Genotypic frequencies of *IGF‐1* gene.

Author	Breed	*N*	Genotypic frequencies
Alex et al. 2023	Attappady Black	50	AA (0.32), TT (0.28), AT (0.4)
	Malabari	50	AA (0.4), TT (0.16), AT (0.44)
Ayele et al. 2024	Saanen		AA (0.033), AB (0.315), BB (0.652)
	Alpine		AA (0.049), AB (0.247), BB (0.704)
	Boer		AA (0.061), AB (0.242), BB (0.697)
Kurdistani et al. 2013	Markhoz	224	GG (0.97), AG (0.03), AA (0)
	Kurdi	72	GG (0.96), AG (0.04), AA (0)
Rasouli et al. 2017	Markhoz	152	GG (0.81), GA (0.16), AA (0.03)
Sarmah et al. 2019	Assam Hill	256	AA (0.19), AB (0.49), BB (0.33)
Shareef et al. 2018	Beetal	60	AA (0.39), AB (0.47), BB (0.09)
Zhang et al. 2008	Nanjiang Huang	592	GG (0.358), GC (0.375), CC (0.267)

### 3.11 Allelic Frequencies of GH Gene

The allelic frequencies that were identified within the selected studies for *GH* gene are presented in Table 5. Three (*n* = 3) studies were clear about their allelic frequencies out of five (*n* = 5) included studies, whereas two (*n* = 2) were not. Six (*n* = 6) allelic frequencies were noted within these three studies. These allelic frequencies ranged from 0.11 to 0.917. Allele B and T showed a great proportion for *GH* gene within the populations of different studies.

**TABLE 5:** Allelic frequencies of *GH* gene.

Author	Breed	*N*	Allelic frequencies
Ayele et al. 2024	Saanen	203	A (0.438) B (0.562)
	Alpine		A (0.444) B (0.556)
	Boer		A (0.197) B (0.803)
Rashijane et al. 2022	Boer	76	A (0.89) B (0.11)
Sharma et al. 2013	Sirohi	389	T (0.917) C (0.082)
	Jamunapari		T (0.878) C (0.121)

### 3.12 Allelic Frequencies of IGF‐1 Gene

The allelic frequencies that were identified within the selected studies for *IGF‐1* gene are presented in Table 6. Seven (*n* = 7) studies were clear about their allelic frequencies out of eight (*n* = 8) included studies, whereas one (*n* = 1) was not. Eleven (*n* = 11) allelic frequencies were noted within the seven studies that included *IGF‐1* gene. These allelic frequencies ranged from 0.02 to 0.98. For genotypes AT, AB and AG, alleles A, B and G, respectively, showed a great proportion for *IGF‐1* gene within the populations of the included articles.

**TABLE 6:** Allelic frequencies of *IGF‐1* gene.

Author	Breed	*N*	Allelic frequencies
Alex et al. 2023	Attappady Black	50	A (0.52) T (0.48)
	Malabari	50	A (0.62) T (0.38)
Ayele et al. 2024	Saanen	—	A (0.191) B (0.809)
	Alpine	—	A (0.173) B (0.827)
	Boer	—	A (0.182) B (0.818)
Kurdistani et al. 2013	Markhoz	224	G (0.98) A (0.02)
	Kurdi	72	G (0.98) A (0.02)
Rasouli et al. 2017	Markhoz	152	G (0.89) A (0.11)
Sarmah et al. 2019	Assam Hill	256	A (0.43) B (0.57)
Shareef et al. 2018	Beetal	60	A (0.65) B (0.)35
Zhang et al. 2008	Nanjiang Huang	592	G (0.546) C (0.454)

### 3.13 Association Between GH Genotypes and Growth Traits

The findings of the *GH* genotypes association with growth traits are shown in Table 7. Five (*n* = 5) studies investigated the association between *GH* gene and growth traits. A total of nine growth traits, including BW, withers height (WH), heart girth (HG), body length (BL), chest width (CW), rump height (RH), rump width (RW), ear length (EL) and cannon circumference (CC) were investigated within the reviewed articles. All five reviewed articles investigated BW; four (*n* = 4) of them found a significant association between genotypes and BW, whereas two found a non‐significant association. Three (*n* = 3) articles investigated WH, with two non‐significant and one significantly associated with genotypes. Five articles investigated HG, with two non‐significant and three significantly associated with genotypes. Four articles found a non‐significant association, whereas one found a significant association between BL and genotypes. Two studies discovered a non‐significant association between CW and genotypes, whereas one found a significant association. One article investigated RH, RW, EL and CC and found no significant association between all the genotypes and traits investigated. Most investigations showed significant associations between *GH* genotypes and growth traits of goats.

**TABLE 7:** *GH* gene SNPs association with growth traits.

Author	Breed	Growth traits	Genotypes			Significance
An et al. 2010	Boer	BW, WH, HG, BL	AA	AB	AC	*
Ayele et al. 2024	Saanen, Alpine	BW, WH, BL, HG	AB	BB		ns
		CW	AB	BB		*
	Boer	BW, HG	AB	BB		*
		WH, BL, CW	AB	BB		ns
Hua et al. 2009	Boer	BW, BL, HG	AA	AB		ns
Rashijane et al. 2022	Boer	BW	AA	AB		*
		BL, HG, RH, RW, EL, CC, WH	AA	AB		ns
Sharma et al. 2013	Sirohi, Jamunapari	BW	AA	AT	TT	*

Abbreviations: BL = body length, BW = body weight, CC = cannon circumference, CW = chest width, EL = ear length, HG = heart girth, HW = head width, RH = rump height, RW = rump width, WH = withers height.

*Significant at *p* < 0.05, ns = non‐significant at *p* > 0.05.

### 3.14 Association Between IGF‐1 Genotypes and Growth Traits

The results of the *IGF‐1* genotype association with growth traits are shown in Table 8. Eight (*n* = 8) studies investigated the association between *IGF‐1* gene and growth traits. Six of the reviewed articles showed a non‐significant association between BW and genotypes, whereas four showed a significant association. Five studies investigated and discovered no significant association between WH, BL and HG with genotypes. Only three studies investigated CW, two of them found no significant association, whereas one found a significant association between the trait and genotypes. Most studies discovered a non‐significant association between *IGF‐1* genotypes and growth traits of goats.

**TABLE 8:** *IGF‐1* gene SNPs association with growth traits.

Author	Breed	Growth traits	Genotypes			Significance
Alex et al. 2023	Attappady Black, Malabari	BW	AA	TT	AT	*
Ayele et al. 2024	Saanen	BW, WH, BL, HG, CW	AA	AB	BB	ns
	Alpine	BW	AA	AB	BB	*
		WH, BL, HG, CW	AA	AB	BB	ns
	Boer	CW	AA	AB	BB	*
		BW, WH, BL, HG	AA	AB	BB	ns
Kurdistani et al. 2013	Markhoz	BW	GG	AG	AA	ns
Rasouli et al. 2017	Markhoz	BW	GG	GA	AA	ns
Sarmah et al. 2019	Assam Hill	BW	AA	AB	BB	ns
Shareef et al. 2018	Beetal	BW, WH, BL, HG	AA	AB	BB	ns
Sharma et al. 2013	Sirohi, Jamunapari	BW	CC	TC	TT	*
Zhang et al. 2008	Nanjiang Huang	BW	GG	GC	CC	*
		BL, WH, HG	GG	GC	CC	ns

Abbreviations: BL = body length, BW = body weight, CC = cannon circumference, CW = chest width, EL = ear length, HG = heart girth, HW = head width, RH = rump height, RW = rump width, WH = withers height.

*Significant at *p* < 0.05, ns = non‐significant at *p* > 0.05.

## 4 Discussion

Growth traits are physiological functions under the control of several genes. *GH* and *IGF‐1* genes operate in coordination to regulate the growth performance of livestock (Angel et al. 2018). This study was conducted to provide information on the association of SNPs of the *GH* and *IGF‐ 1* genes and growth traits of goats. *GH* gene influences the growth of bones and muscles, which is mediated by *IGF‐1* gene, which is a key factor for the postnatal growth of goats (Alex et al. 2023). A comprehensive understanding of goat growth and development and the factors influencing these processes is crucial because it impacts production efficiency and directly impacts product quality (Webb 2014).

The results of this systematic review indicated that one article of Kurdistani et al. (2013) found two SNPs (1617 G>A and 5752 G>C) that were similar to the ones of two other articles each for *GH* gene. Rasouli et al. (2017) also found SNP 1617 G>A; SNP 5752 G>C was also discovered by Sarmah et al. (2019). All the other articles discovered different SNPs for both *IGF‐1* gene and *GH* gene. The association between *GH* gene and growth traits of goats was investigated by five articles. An et al. (2010) discovered that these SNPs (112A>G, 142C>T, 214C>T and 266C>A) were associated with all the growth traits investigated of Saanen and Boer goats. Sharma et al. (2013) and Rashijane et al. (2022) found an association between BW and SNPs (4700T>C and 5524C>T) and SNP (505G>C) of Sirohi and Jamunapari and Boer goats, respectively. SNP 505G>C was also found to be not associated with all the other investigated growth traits of Boer goats. These SNPs (781A>G and 1575A>G) were also found to be not associated with the growth traits of Boer goats (Hua et al. 2009).

The association between the *IGF‐1* gene and growth traits of goats was investigated by eight articles. Zhang et al. (2008), Sharma et al. (2013) and Alex et al. (2023) reported that BW was associated with SNP (589G>C), SNPs (4700T>C and 5524C>T) and SNP (c.546þ179170A>T) of Nanjiang Huang, Sirohi and Jamunapari and Attappady Black and Malabari goat breeds, respectively. SNP (589G>C) was also found to be not associated with any other growth traits except BW of Nanjiang Huang goat breed (Zhang et al. 2008). Kurdistani et al. (2013), Rasouli et al. (2017), Shareef et al. (2018) and Sarmah et al. (2019) reported that BW had no association with SNPs (1617 G>A and 5752 G>C), SNP (1617 G>A), SNP (825A>G) and SNP (5752G>C) of Markhoz and Kurdi, Markhoz, Beetal and Assam Hill goat breeds, respectively. SNP (825A>G) was also found to not be associated with any other growth traits investigated (Shareef et al. 2018). The difference between the results in different articles might be due to the different environments, breeds and management systems used in the studies.

To the best of our knowledge, this is the first systematic review reporting associations between *GH* and *IGF‐1* gene SNPs and goat growth traits. The results of this systematic review imply that some of the goats’ *GH* and *IGF‐1* genes’ SNPs may be used as a genetic marker for the selection of growth traits of goats, such as BW and CW for *IGF‐1* gene and BW, WH, HG, BL and CW for *GH* gene. The contribution of this systematic review to the body of knowledge is that some of the identified goats’ *IGF‐1* and *GH* gene SNPs are associated with some growth traits while others are not associated. The limitation of this study is that only two common SNPs were identified, and they were only identified by two articles each. It is thus not possible to conduct the meta‐analysis for this data. Hence, it is highly recommended that more studies be conducted for the identification of *GH* and *IGF‐1* genes’ SNPs and their association with the growth traits of goats.

## 5 Conclusion

This systematic review concludes that the *IGF‐1* gene did not have an influence on WH, BL and HG, but had an influence on BW and CW. The *GH* gene did not influence RH, RW, EL and CC, but had an influence on BW, WH, HG and BL. SNPs of *IGF‐1* and *GH* that showed association with traits may serve as potential molecular markers for the genetic improvement of these traits in goats breeding.

## Author Contributions

**Thlarihani Cynthia Makamu**: writing – review and editing, writing – original draft. **Thobela Louis Tyasi**: writing – review and editing, writing – original draft, supervision. **Monnye Mabelebele**: writing – review and editing, writing – original draft, supervision.

## Funding

This systematic review was financially supported by the South African National Research Foundation (postgraduate: reference no. PMDS230621119261).

## Ethics Statement

Permission to conduct this study in terms of Section 20 of Animal Diseases Act, 1984 (Act No. 35 of 1984), was granted by the Department of Agriculture, Land Reform and Rural Development with reference number: 12/11/1/1/23/17110/(SP). Ethical clearance with reference number 2024/CAES_AREC/6014 was obtained from the University of South Africa College of Agriculture and Environmental Sciences _Animal REC.

## Conflicts of Interest

The authors declare no conflicts of interest.

## Data Availability

All data generated during this study are available through a request to the corresponding author.
